# Differential Analysis of Gly211Val and Gly286Val Mutations Affecting Sarco(endo)plasmic Reticulum Ca^2+^-ATPase (SERCA1) in Congenital Pseudomyotonia Romagnola Cattle

**DOI:** 10.3390/ijms232012364

**Published:** 2022-10-15

**Authors:** Eylem Emek Akyürek, Francesca Busato, Leonardo Murgiano, Elisa Bianchini, Marcello Carotti, Dorianna Sandonà, Cord Drögemüller, Arcangelo Gentile, Roberta Sacchetto

**Affiliations:** 1 Department of Comparative Biomedicine and Food Science, University of Padova, Viale dell’Università 16, Legnaro, 35020 Padova, Italy; 2 Veterinary Clinic San Marco, Viale dell’Industria 3, Veggiano, 35030 Padova, Italy; 3 Department of Clinical Sciences and Advanced Medicine, School of Veterinary Medicine, University of Pennsylvania, 3900 Delancey Street, Philadelphia, PA 19104, USA; 4 Department of Biomedical Sciences, University of Padova, Via U. Bassi 58/b, 35131 Padova, Italy; 5 Aptuit, Via A. Fleming 4, 37135 Verona, Italy; 6 Institute of Genetics, Vetsuisse Faculty, University of Bern, Bremgartenstrasse 109a, 3012 Bern, Switzerland; 7 Department of Veterinary Medical Sciences, University of Bologna, Via Tolara di Sopra 50, 40064 Ozzano Emilia, Italy

**Keywords:** skeletal muscle diseases, cattle congenital pseudomyotonia, human Brody disease, sarco(endo)plasmic reticulum Ca^2+^ ATPase isoform 1 (SERCA1), *ATP2A1* gene

## Abstract

Congenital pseudomyotonia in cattle (PMT) is a rare skeletal muscle disorder, clinically characterized by stiffness and by delayed muscle relaxation after exercise. Muscle relaxation impairment is due to defective content of the Sarco(endo)plasmic Reticulum Ca^2+^ ATPase isoform 1 (SERCA1) protein, caused by missense mutations in the *ATP2A1* gene. PMT represents the only mammalian model of human Brody myopathy. In the *Romagnola* breed, two missense variants occurring in the same allele were described, leading to Gly211Val and Gly286Val (G211V/G286V) substitutions. In this study, we analyzed the consequences of G211V and G286V mutations. Results support that the reduced amount of SERCA1 is a consequence of the G211V mutation, the G286V mutation almost being benign and the ubiquitin–proteasome system (UPS) being involved. After blocking the proteasome using a proteasome inhibitor, we found that the G211V mutant accumulates in cells at levels comparable to those of WT SERCA1. Our conclusion is that G211/286V mutations presumably originate in a folding-defective SERCA1 protein, recognized and diverted to degradation by UPS, although still catalytically functional, and that the main role is played by G211V mutation. Rescue of mutated SERCA1 to the sarcoplasmic reticulum membrane can re-establish resting cytosolic Ca^2+^ concentration and prevent the appearance of pathological signs, paving the way for a possible therapeutic approach against Brody disease.

## 1. Introduction

Congenital pseudomyotonia in cattle (PMT) is a rare skeletal muscle disorder with a recessive mechanism of inheritance. It is clinically characterized by stiffness and progressive impairment of muscle relaxation induced by exercise [1]. Cattle PMT was first described in the *Chianina* breed [1], and at almost the same time, in the *Belgian Blue* breed (termed congenital muscular dystonia1 or CMD1) [2]. Subsequently, it was found in the *Romagnola* cattle breed [3] and as a single case in a *Dutch Improved Red and White* (DRW) calf [4]. It has been well established that the muscle relaxation impairment underlying cattle PMT is due to defective content in the muscles of Sarco(endo)plasmic Reticulum Ca^2+^ ATPase isoform 1 (SERCA1), caused by missense variants in the *ATP2A1* gene [5]. In vertebrates, three genes (*ATP2A1-3*) encode three SERCA1 isoforms, differentially expressed in a tissue-dependent fashion and according to the stage of development [6]. SERCA1 is the main protein component of the non-junctional sarcoplasmic reticulum (SR) membranes of adult fast-twitch (type 2) skeletal muscle fibers. At the end of the contraction cycle, SERCA1 transports two Ca^2+^ ions from cytosol into the SR lumen at the expense of the hydrolysis of one ATP molecule, thus initiating muscle relaxation [7].

In *Chianina*, as well as *Dutch Improved Red White* and *Belgian Blue*, affected animals, DNA sequencing detected single causative homozygous missense mutations in SERCA1. In *Chianina*, the causative mutation replaces an Arg at position 164 by a His (R164H). In *Dutch Improved Red White*, as well as in *Belgian Blue*, the variant leads to an Arg559Cys (R559C) substitution in a highly conserved domain of the protein. In the *Romagnola* breed, two missense mutations in exon 8 (occurring in the same allele) were described, leading to Gly211Val and Gly286Val (G211V and G286V) substitutions. Most *Romagnola* PMT-affected calves are in fact compound heterozygous, carrying the two point mutations in one allele, and the R164H mutation already found in the *Chianina* breed, in the other allele. Only one PMT *Romagnola* subject was found homozygous for the G211V/G286V variant [3], found exclusively in this breed.

Irrespective of the type of genetic variant affecting the *ATP2A1* gene, a striking selective reduction of SERCA1 mutant protein expression has been observed in SR membranes isolated from PMT-affected bovine muscles from all different breeds [3,4,8]. In *Chianina*, this was because R164H SERCA1 amino acid substitution leads to a protein defective in proper folding, as we recently clarified [9]. The R164H mutant, although still functionally active, is recognized as corrupted and prematurely degraded by the ubiquitin–proteasome system (UPS). Given the decrease in SERCA1 pump at the SR membrane, the re-uptake of calcium and the lowering of its cytosolic concentration slowed down, triggering muscle contracture. This study is focused on the G211V/G286V mutations exclusively found in the *Romagnola* breed. We tested whether one or both amino acid substitutions would play a major role in causing the reduced expression level of the SERCA1 protein and whether, in the same way as the R164H mutant, the UPS is involved in the reduced expression at the SR membrane of this SERCA1 mutant, causing PMT in *Romagnola* cattle.

## 2. Results

### 2.1. Effect of G211V and G286V Mutations on SERCA1 Protein Expression

To investigate the role of the G211V/G286V amino acid substitutions in causing the reduced expression of the SERCA1 protein, three different cDNA constructs were created. Rabbit full-length Ca^2+^ ATPase isoform cDNA was the first generated SERCA1 construct [10], widely used in cellular expression, enzymatic function and site-directed mutagenesis studies [11], including *Chianina* R164H mutation investigations [9]. In this study, SERCA1 mutations were introduced in the full-length cDNA encoding the adult isoform of fast-twitch SERCA1 of bovine origin. Using the site-directed mutagenesis technique, mutations were introduced together (G211/286V as cDNA double mutant), resembling the condition found in PMT-affected animals, or separately, generating both G211V and G286V variants, respectively. Mutant vectors and the WT form were then transfected in HEK293 cells and SERCA1 expression was assessed by immunofluorescence analysis.

**Figure 1:**
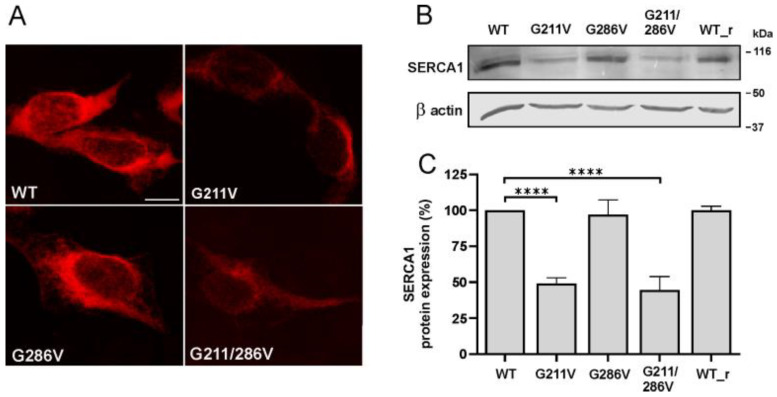
Cellular localization and expression level analysis of SERCA1 protein in HEK293 cells transfected with WT and mutant SERCA1 constructs. Cells were transfected with WT SERCA1 cDNA of bovine or rabbit (WT_r) origin, or with G211V, G286V and G211/28164H-mutated SERCA1 cDNAs, as indicated. (**A**) Transfected cells were immunolabelled with monoclonal antibodies to SERCA1 and then incubated with the appropriate secondary antibody (red fluorescence). Images were recorded at the same setting conditions and magnification (scale bar 25 μm). (**B**) Total protein lysates from cells transfected with WT or mutated SERCA cDNAs were obtained by solubilization. An equal quantity of protein was separated by SDS-PAGE and blotted onto nitrocellulose paper. The blots were incubated with antibodies specific for SERCA1 and the 42 kDa beta-actin, used as a loading control. A representative Western blot is shown. (**C**) Quantification of SERCA1 protein bands performed by densitometric analysis on Western blots. Data (mean values from at least four independent experiments ± S.D.) are reported as the percentage of values from WT SERCA1 of bovine origin. Statistical analysis was performed by the one-way ANOVA test, followed by Dunnett’s multiple comparisons test. **** *p* ≤ 0.0001.

Figure 1A describes the immunostaining pattern of the anti SERCA1 antibody in cells expressing the cDNA constructs. In all cases, the typical staining pattern of endoplasmic reticulum (ER) proteins was observed, a thin and diffuse network distributed throughout the cytoplasm. Co-localization using anti calreticulin antibodies, a well-known ER marker, confirmed that SERCA1 mutants are correctly targeted to ER (Figure S1). Figure 1A also shows that the expression level of both SERCA1 G211V and G211/286V variants was qualitatively equivalent, but remarkably lower than WT. On the other hand, when the cDNA construct carrying the single G286V variant was used, the immunofluorescence pattern was found to be very similar to that of WT.

These experiments were paralleled by immunoblot analysis on total HEK293 cell lysate with the same specific antibody against SERCA1. Results reported in Figure 1B,C, confirm the existence of wide differences in the expression level of SERCA1 variants, depending on the mutations introduced. The expression level of both the single-mutant G211V and the double-mutant G211/286V SERCA1 were lower than the WT, in accor-dance with what is observed by immunofluorescence in Figure 1A. Vice versa, when the G286V cDNA was expressed, the level of SERCA1 mutant was comparable with that of the WT. The result obtained with the double-mutant G211/286V corresponded to what was previously found in pathological muscle fibers from PMT bovine patients, as shown in our previous work [3,12] and in Figure S2. Figure S2 also shows that, despite the reduction in immunoreactivity observed in pathological muscle transverse sections immunolabelled with anti SERCA1 antibodies, no morphological difference was observed between samples from both homozygous (G211V/G286V) and heterozygous (G211V/G286V and R164H) PMT and normal muscle samples.

### 2.2. Functional Comparison of Cells Transfected with Different Vectors Expressing G211/286V, G211V and G286V Mutants

**Figure 2:**
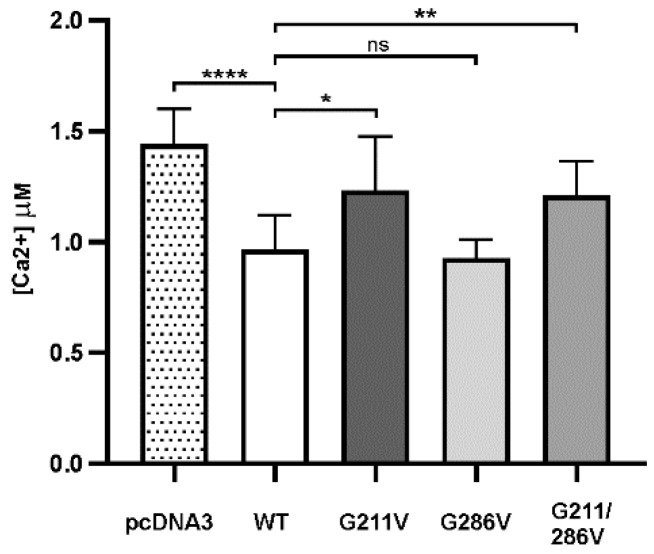
Cytosolic calcium measurements in cells expressing the WT and mutant SERCA1 variants. The plasmid expressing the cytosolic Ca^2+^-sensitive photoprotein aequorin (CytAEQ) was co-transfected in HEK293 cells with either WT, G211V, G286V, G211/286V SERCA1 cDNAs or with the empty vector (pcDNA3), as indicated. Cytoplasmic Ca^2+^ transients were measured after agonist stimulation (carbachol 500 μM). The histogram shows the mean values ± SD of Ca^2+^ transient peaks from six independent experiments. Statistical analysis, referred to cells expressing the WT form, was performed by the one-way ANOVA test, followed by Dunnett’s multiple comparisons test. **** *p* ≤ 0.0001; * *p* ≤ 0.05; ** *p* ≤ 0.01.

HEK293 cells transfected with WT or mutant plasmids for SERCA1 were then co-transfected with the cytosolic Ca^2+^-sensing photoprotein aequorin (cytAEQ) and stimulated with carbachol, the inositol 1,4-5 agonist that induces an increase in cytoplasmic Ca^2+^ due to its release from the ER, as well as Ca^2+^ influx across the plasma membrane [13]. As previously reported [9], cells transfected with WT SERCA1 were found to significantly accumulate Ca^2+^ after stimulation induced Ca^2+^ transients (Figure 2). The same Figure 2 also shows that cells overexpressing SERCA1 pump carrying the G211V mutation (alone or combined with G286V) had a much lower Ca^2+^ pumping ability compared to the WT. This was demonstrated by the enhanced height of the peak of the Ca^2+^ transient, almost equal to that of cells transfected with the empty vector (pcDNA3). The enhanced height of the Ca^2+^ transient peak also reflected a prolonged retention of Ca^2+^ within the cytoplasm, indicating that Ca^2+^ re-uptake is slowed down [13]. This is in close agreement with the decreased density of both G211V and G211/286V mutant pumps. In a very surprising way, we observed that cells overexpressing the G286V mutation alone exhibit the same ability as the WT form to re-accumulate Ca^2+^ from the cytosol.

### 2.3. SERCA1 mRNA Expression Levels in Control and PMT-Affected Calf Muscles and Functional Comparison of Ca^2+^ ATPase Activities on Isolated Skeletal Muscle SR Membranes

**Figure 3:**
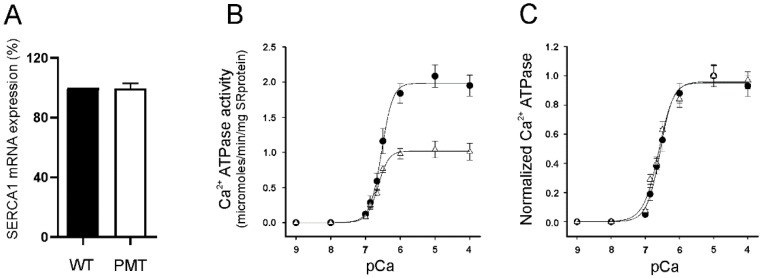
Expression levels of SERCA1 mRNA and calcium-dependent ATPase activity in controls and PMT-affected muscles carrying G211V/G286V SERCA1 mutations. (**A**) SERCA1 mRNA expression levels in healthy control (WT) and PMT-affected (PMT) calf muscles were quantified by RT-PCR. Data are reported as the relative expression relative to control muscles. Data were obtained from different mRNA extractions from the same pathological muscle specimen and are reported as the mean expression values ± SD. (**B**) The Ca^2+^-dependent ATPase activity was evaluated using an enzyme-linked ATPase assay performed on SR microsomes isolated from homozygous G211V/G286V *Romagnola* PMT calf and from unaffected *Romagnola* muscle specimens. The Ca^2+^ sensitivity of the homozygous PMT calf was comparable with that of the WT. Data, expressed as micromoles/min/mg SR protein, are the mean ± S.D. Symbols: black circle: control animals; white triangle: G211V/G286V PMT-affected calf. (**C**) The specific ATPase activity was normalized based on the relative content of SERCA1 protein calculated by densitometric measurements conducted on immunoblots. Symbols: black circle: control animals; white triangle: G211V/G286V PMT-affected calf.

Before analyzing the Ca^2+^ ATPase activity in skeletal muscle, we investigated the levels of SERCA1 mRNA. Real-time PCR experiments performed to investigate whether G211V/G286V mutations on the *ATP2A1* gene affect the mRNA transcript and its stability showed comparable expression between pathological and control samples (Figure 3A).

Ca^2+^ ATPase activity was next investigated using an enzyme-linked ATPase assay carried out on microsomal fractions enriched in content of SR membrane. The membranes were isolated from muscles of the unique homozygous G211V/G286V *Romagnola* PMT calf (Figure 3B,C). In our previous work [3], we reported that SERCA1 Ca^2+^ ATPase activity measured at optimum pCa (pCa5) was decreased in SR membrane fractions from both heterozygous (G211V/G286V and R164H) and homozygous (G211V/G286V) PMT-affected *Romagnola* cattle compared to healthy animals. Figure 3B shows a comparison of the Ca^2+^ dependence of Ca^2+^ ATPase activity of the G211V/G286V mutant exclusively found in the *Romagnola* breed, versus the WT. Results clearly show that, although the pCa dependence was almost identical (pKCa 0.5 values of 6.59 and 6.64 for WT and pathological membrane samples, respectively), the levels of ATPase activity (expressed as mg of SR protein) were much lower in the double mutant. However, when the levels of ATPase activity were normalized for the content of SERCA1 protein in SR membranes [14], the ATPase activities of WT and G211V/G286V mutants (Figure 3C) were found to be so close that their trends were almost superimposable.

### 2.4. Quantitative and Qualitative Recovery of G211V SERCA1 Mutant after Proteasome Inhibition and Ubiquitination of G211V Mutant

**Figure 4:**
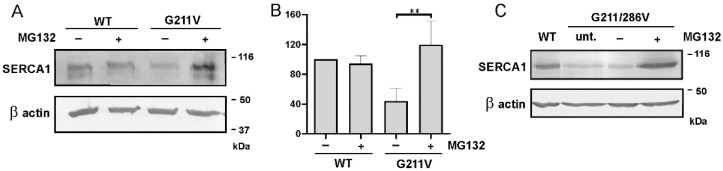
Expression level analysis of WT, G211V and G211/286V mutant SERCA1 proteins after incubation with proteasome inhibitor MG132. (**A**) HEK293 cells transfected with WT and G211V SERCA1 cDNAs were treated with the proteasome inhibitor MG132 (10 μM) (+) or its vehicle DMSO (0.1%) (−). An equal quantity of protein from total-cell lysates was separated by SDS-PAGE and subjected to immunoblot analysis with antibodies specific to SERCA1 and 42 kDa beta-actin used as the loading control. A representative Western blot is shown. (**B**) The graph shows quantification of SERCA1 protein bands performed by densitometric analysis on Western blots. Data (mean values from at least four independent experiments ± S.D.) are reported as the percentage of values from the WT. Statistical analysis was performed by unpaired two-tailed Student’s *t*-test. ** *p* ≤ 0.05. (**C**) HEK293 cells were transfected with WT and G211/286V SERCA1 cDNAs. Cells transfected with the mutant plasmid were then untreated (unt.) or treated with the proteasome inhibitor MG132 (10 μM) (+) or its vehicle DMSO (0.1%) (−) and subjected to immunoblot analysis, as described above.

To investigate the possible involvement of the UPS in the decreased protein level of SERCA1 carrying the G211V mutation, HEK293 cells transfected with the G211V plasmid were incubated with MG132, a well-known proteasome inhibitor. Immunoblot analysis of total-cell lysate with antibodies to SERCA1 (Figure 4A) revealed that, after eight hours of MG132 treatment, the protein level of the G211V mutant is significantly increased, while, as expected, the WT protein expression was virtually unaffected. An identical result was obtained using the double-mutant G211/286V (Figure 4C).

**Figure 5:**
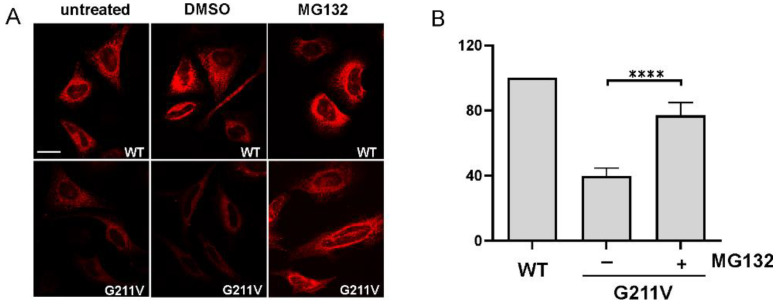
Restoration of expression level and Ca^2+^ ATPase activity of G211V-mutated SERCA1 protein after incubation with proteasome inhibitor MG132. (**A**) HeLa cells were transfected with WT or G211V SERCA1 cDNAs. Cells were then treated with the proteasome inhibitor MG132 (10 μM) or its vehicle DMSO (0.1%), as indicated. For comparison, untreated cells expressing the WT and G211V mutant were also analyzed. SERCA1 expression was evaluated by confocal immunofluorescence analysis of cells immuno-decorated with a monoclonal antibody to SERCA1 and then incubated with the appropriate secondary antibody (red fluorescence). Images were recorded at the same setting conditions and magnification (scale bar 50 μm). (**B**) A microsomal membrane fraction was isolated from HEK293 cells transfected with WT or G211V SERCA1 cDNAs. Mutant plasmid-transfected cells were treated with the proteasome inhibitor MG132 (10 μM) (+) or its vehicle DMSO (0.1%) (−). Ca^2+^ ATPase activity of microsomal fraction was determined by a spectrophotometric enzyme-coupled assay. Data (mean values from three independent experiments ± S.D.) are reported as the percentage of values from the WT. Statistical analysis was performed by unpaired two-tailed Student’s *t*-test. **** *p* ≤ 0.0001.

Immunoblot findings reported in Figure 4A were confirmed by immunofluorescent analysis on cells transfected with G211V SERCA1 plasmid (Figure 5A). A very low amount of G211V SERCA1 mutant was detected in both untreated and DMSO-treated cells. After incubation with MG132, the intensity of the signal becomes almost equal to the one found in WT cells.

Ubiquitination is the first step for proteins destined for degradation via the UPS. Ubiquitin pull-down assay was performed to investigate G211V SERCA1 polyubiquitination. Immunoblot analysis with an anti-ubiquitin antibody of immunocomplexes showed that the SERCA1-specific antibody recovered the G211V SERCA1 in the form of a complex with ubiquitin. Inhibition of the UPS with MG132 led to an increase in the recovery of the polyubiquitinated G211V mutant, as hypothesized (Figure S3).

**Figure 6:**
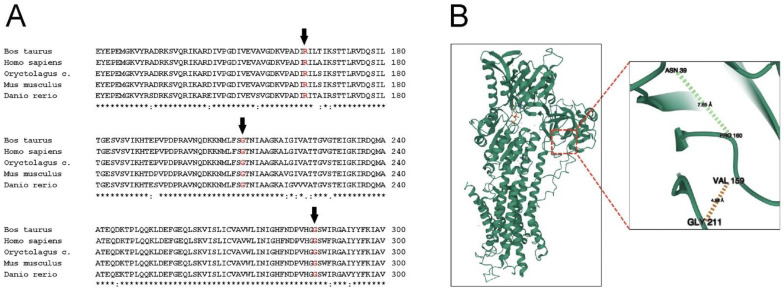
Cross-species SERCA1 amino acid sequence comparison and graphic representation of SERCA1 protein of bovine origin (PDB ID 3TLM). (**A**) Multiple sequence alignment of bovine SERCA1 with its orthologs. Only the sequence around the R164H and G211V/G286V mutations, found in *Chianina*, as well as *Romagnola*, and exclusively in *Romagnola* cattle breeds, is shown respectively. Amino acid substitutions are indicated by arrows. A high degree of conservation of amino acid residues is shown to occur not only across mammals but also between zebrafish and mammalian SERCA1 sequences. (**B**) Detail of domain A with the Gly211 amino acid (pathogenic in *Romagnola*) is shown.

SERCA1 G211V rescued by treatment with MG132 was evaluated for its functional properties. The Ca^2+^ ATPase activity at optimum pCa (pCa5) was assayed on microsomal membrane fraction isolated from HEK293-transfected cells, using an enzyme-linked ATPase method. Microsomes from cells transfected with G211V SERCA1 displayed a Ca^2+^ ATPase activity lower than that of microsomes from WT-transfected cells (Figure 6), in agreement with results obtained using SR microsomal fraction from pathological muscles (Figure 3B). On the other hand, incubation of G211V-transfected cells with proteasome inhibitor MG132 led to a significant increase in microsomal Ca^2+^ ATPase activity (Figure 5B). This finding correlates with the increased expression of the mutant pump and strongly suggests that G211V mutation does not abolish SERCA1 pump activity.

## 3. Discussion

Bovine PMT is a rare muscular disorder described for the first time in *Chianina* cattle [1]. PMT pathophysiology has been fully clarified in this breed [9]. We provided unequivocal evidence that the SERCA1 R164H mutation generates a protein most likely to be affected in its proper folding. Although still functionally active, the protein was ubiquitinated and removed by the UPS. The relevance of these studies, carried out on cattle PMT, remains in its similarity to human Brody disease, a rare autosomal recessive myopathy, characterized by muscle cramps and stiffness occurring after muscular activity or even mild exercise (such as climbing the stairs) [15]. As in the case of bovine PMT, Brody disease is due to SERCA1 deficiency [16], resulting from defects of the *ATP2A1* gene [17], many of which are missense mutations [18]. At present, a mouse model is not available for Brody disease. For these reasons, bovine PMT represents the only and widely accepted mammalian model for studying Brody disease.

Brody myopathy is also an orphan disease, since a specific therapy, to date, does not exist. Indeed, Brody patients are usually treated with generic muscle relaxant drugs that prevent calcium release from the SR [19]. However, these molecules are unsuitable for long-term treatments and the therapy is often suspended due to side effects [18]. The fact that the functional characteristics of the enzyme are preserved represents the fundamental prerequisite for any possible innovative pharmacological therapy based on the recovery of normal protein levels of SERCA1.

Bovine PMT has been more recently described in *Romagnola* cattle. In a previous work, we reported that, in this breed, PMT missense mutations in the *ATP2A1* gene lead to G211V/G286V substitutions in the SERCA1 protein, in addition to the R164H substitution already described in *Chianina* PMT (pathogenic SERCA1 variants described thus far in bovine species are summarized Table S1). Compound heterozygosis is frequently reported in PMT-affected *Romagnola*, except for one pathological case that was found to be homozygous for the novel complex variant G211V/G286V. Heterozygosity is most likely due to the accidental introgression of *Chianina* PMT-affected animals in the ancestry of the *Romagnola* PMT founder sire. Independently from differences in genotype, skeletal muscles of *Romagnola* PMT cattle are characterized by a selective low expression level of the SERCA1 protein in SR membranes [3], as found in *Chianina* PMT.

Here, we analyzed the relative impact of the G211V and G286V mutations, exclusively found in the *Romagnola* breed. We used an experimental approach involving a heterologous cellular system transfected with the bovine SERCA1 expression constructs carrying G211/286V mutations together or independently. Our results clearly show that, of the two variants, G211V is the pathogenic one. In fact, the reduction in SERCA1 content, as well as in Ca^2+^ pumping and Ca^2+^ ATPase activity of SR membranes, is virtually identical when G211V is expressed alone or in combination with G286V. Vice versa, the G286V mutant does not show any difference with the WT in regard to those parameters.

In our previous work, we reported that the reduction in SERCA1 pump Ca^2+^ ATPase activity in SR membranes from PMT *Romagnola* muscles consistently correlated with the decreased protein expression in both heterozygous and homozygous subjects [3,12]. The results described here using both transfected cells and transverse cryosections from pathological skeletal muscles demonstrate that the G211V mutation does not interfere with proper localization of mutated SERCA1 pump. Using SR membrane fraction from the unique homozygous bovine patient (it must be remembered that PMT is a rare disease), we have clearly shown that the Ca^2+^ dependence of the Ca^2+^ ATPase activity was maintained, the pKCa50 value being almost identical to that of the WT SERCA1 protein (Figure 3B,C). Moreover, the Ca^2+^ ATPase activity in microsomes isolated from cells transfected with G211V SERCA1 and treated with the proteasome inhibitor was found to increase in correlation with the accumulation of this mutant protein within the ER membranes. Overall, these data indicate that the G211V mutation, although responsible for the reduced expression of the mutant SERCA1 protein, does not affect its catalytic function. Since in muscle specimens the mutation does not alter the transcription levels of the *ATP2A1* gene, the reduction in content of the SERCA1 protein both in cells and muscle [3,12] must reflect a posttranslational event. Our conclusion is that, as for the R164H SERCA1 mutant causing PMT in *Chianina* cattle [9], G211V/G286V mutations found in *Romagnola* presumably originate in a folding-defective SERCA1 protein that is recognized and diverted to degradation by the UPS, while still being catalytically functional. Furthermore, in this event, the main role is played by the G211V mutation, the other variant, G286V, being almost ineffective.

Gly211 residue is located in the actuator (A) domain of SERCA1. The bovine SERCA1 molecular model, very similar to that of the rabbit enzyme, as expected by the high amino acid sequence identity [20], revealed that Gly211 is surrounded by Val159 Pro160 residues, in addition to Asn39 (Figure 6). This is in close agreement with that found by Miyauchi and colleagues [21], who used the atomic structure of rabbit SERCA1. In *Romagnola* PMT, the single neutral hydrogen atom side chain of Gly211 is substituted with the bulky aliphatic hydrophobic side chain of Val. It is conceivable that this group, which tends not to form hydrogen or ionic bonds with other groups in near proximity, interferes with side-chain interactions of surrounding residues, moving them away and ultimately leading to destabilization of the structure. It is possible to speculate that this substitution might be the cause of folding alteration. This is not surprising, since substitution of Gly211 residue in SERCA2b, the major isoform expressed in keratinocytes, has been depicted as one of the several mutations causing Darier disease, a skin disorder characterized by keratosis [22]. The SERCA2b “house-keeping” variant and SERCA1 adult isoform selectively expressed in fast-twitch type II skeletal muscle fibers, although differentially expressed, share a high degree of homology [6]. On the other hand, Gly286 residue is located in the second intraluminal loop of SERCA1, connecting M3 and M4 transmembrane alpha-helix domains [23]. It is plausible that the substitution of the hydrogen atom of Gly with the long-branched side chain of Val was not relevant in perturbing the structure, due to the short length of this loop (almost 10 residues, as calculated by atomic structure) and to its distance from all other functional domains of SERCA1.

Recently, we proposed a pharmacological approach based on the use of CFTR correctors, specifically developed for rescuing type II mutations of the Cystic Fibrosis Transmembrane Regulator (CFTR) (e.g., F508del-CFTR), causing cystic fibrosis [24]. It has been evidenced in vitro, as well as in vivo, that using these small molecules is possible to avoid the degradation of several alpha-sarcoglycan mutants (causing limb-girdle muscular dystrophy type R3, LGMDR3) that were consequently rescued at the plasma membrane level [25,26]. This new therapeutic strategy has also been proposed for Brody disease (European Patent EU 2925317). Cystic fibrosis, LGMDR3 and Brody myopathy, although different in symptoms and outcome, share the same pathogenetic mechanism: the “loss of function” due to the early disappearance of a protein [9,27,28]. Acting to avoid the premature disposal of mutated SERCA1, this therapy could become a pharmacological approach addressing a specific population of Brody patients with documented reduced expression, but not activity, of the SERCA1 pump. Preliminary results obtained in vitro using cell models transiently expressing the R164H SERCA1 mutant are indeed quite encouraging.

As mentioned above, neither specific therapy nor mouse models exist for Brody disease. Interestingly, a SERCA1 knockout mouse was generated [29]. Newborn SERCA1-null mice displayed limb contractures as in Brody human patients [18,29]. However, SERCA1-null mice displayed gasping respiration and severely impaired diaphragm function due to the high percentage of fast-twitch fibers in this muscle. In fact, a wide difference in fiber composition of the diaphragm muscle has been described between mice and large mammals, including humans [29,30,31]. Fast-twitch fibers account for more than 90% of the diaphragm muscle in mice, compared to about 60% in humans. The SERCA1 protein, which is mutated in Brody disease, is exclusively expressed in skeletal muscle fast-twitch fibers. Consequently, newborn mice died by respiratory failure within two hours and the histological examination of the diaphragm revealed a severe hyper contracture injury of the muscle. This result may explain in part why no SERCA1 knockout in mouse models is available to date.

In conclusion, our data argue that reduced quantities of SERCA1 in *Romagnola* PMT-affected cattle were a consequence of the G211V mutation, the G286V mutation being practically ineffective. Moreover, results presented in this paper, together with data already collected for the R164H mutant, show that both SERCA1 point mutations found in *Chianina* and *Romagnola* cattle affect the expression of the protein but not its catalytic activity. Since null mice or natural mutants for Brody disease are not available, we hope that bovine PMT, although alternative and unconventional, might become a valuable tool for validating any pharmacological approaches in vivo, an essential step on the way to developing novel and specific treatment for the human Brody myopathy.

## 4. Materials and Methods

### 4.1. SERCA1 Construct and Site-Directed Mutagenesis

The full-length adult bovine SERCA1 cDNA was synthetically generated by Eurofins Genomics (Ebersberg, Germany), based on the published database sequence of *Bos taurus* (cow) SERCA1 mRNA (GenBank cDNA clone MGC:140007). *Hind*III and *Not*I restriction sites were added upstream of the ATG and downstream of the STOP codon, respectively, to allow the fragment cloning in the pcDNA3.1 expression vector. The G211V, G286V and G211V/G286V SERCA1 substitutions were generated by using the QuikChange site-directed mutagenesis kit (Stratagene, San Diego, CA, USA), according to the manufacturer’s specifications. The mutagenic primers (Eurofins Genomics, Ebersberg, Germany), annealing in the region where the mutation was introduced, were designed with a mismatch centered to the nucleotide to be changed. The construct was verified by sequencing.

### 4.2. Cell Culture, Transfection and Treatment with Proteasome Inhibitor

HEK293 or HeLa cells (ATCC, Manassas, VA, USA) were counted, seeded and grown in DMEM high glucose medium supplemented with 10% FBS. Cells were transfected with wild-type (WT) and G211V, G286V, G211/286V SERCA1 mutant cDNAs, using TransIT-293 (Mirus Bio) or jetOPTIMUS^®^ DNA (Polyplus Transfection, New York, NY, USA) transfection reagents, according to the manufacturer’s instructions. Sixteen hours after transfection, MG132 (Sigma-Aldrich, St. Louis, MO, USA), an inhibitor of the UPS, was added (10 µM final concentration dissolved in DMSO) and cells were incubated for 8 h [8]. After treatment, cells were washed twice with ice-cold phosphate-buffered saline (PBS) and lysed in a buffer containing Tris-HCl 50 mM pH 7.5, NaCl 150 mM and NP40 1% (*v*/*v*), supplemented with protease inhibitors (Sigma-Aldrich, St. Louis, MO, USA). Protein concentrations were determined by the Bicinchoninic Protein Assay Kit (Quantum Protein Assay Kit, EuroClone Pero, MI, Italy).

### 4.3. Gel Electrophoresis and Immunoblotting

Proteins were resolved by sodium dodecyl sulfate–polyacrylamide gel electrophoresis (SDS-PAGE) and transferred onto a nitrocellulose membrane. The blots were exposed for an hour of 3% Bovine serum albumin in Tris-buffered saline, Tween 20 (TBS-T) (TBS + 0.05% Tween-20) and then probed with mouse-monoclonal antibodies against SERCA1 (dilution 1:5000, ThermoFisher Scientific, Waltham, MA, USA) or β-actin (dilution 1:30,000, Sigma Aldrich, St. Louis, MO, USA). The membranes were then incubated with secondary antibody (dilution 1:30,000, Sigma-Aldrich, St. Louis, MO, USA) alkaline phosphatase or horseradish peroxidase conjugated and developed with BCIP/NBT solution or ECL chemiluminescent substrate, respectively. The blots were imaged with iBright 1500 (ThermoFischer Scientific, Waltham, MA, USA). Quantification of protein bands was performed by densitometric analysis on Western blots from at least four independent experiments.

### 4.4. Immunofluorescence Analysis

HEK293 and HeLa cells were grown on 13 mm glass coverslips. After transfection and treatment with proteasome inhibitor, cells were gently washed with PBS pH 7.4 and fixed with 4% paraformaldehyde for 15 min at room temperature. Cells were washed with PBS and then permeabilized in 0.5% Triton X-100 in PBS. As primary antibodies, mouse monoclonal against SERCA1 (dilution 1:500, ThermoFisher Scientific, Waltham, MA, USA) and rabbit polyclonal against calreticulin (dilution 1:200, Enzo LifeScience, New York, NY, USA) were used in 1% BSA/PBS solution. After the incubation period, cells were gently washed with PBS and incubated with the Alexa Fluor 568 red or Alexa Fluor 488 green secondary antibody (dilution 2 μg/mL, ThermoFisher Scientific, Waltham, MA, USA). Nuclear morphology was characterized by staining with Hoechst 33342 (dilution 1 μg/mL, ThermoFisher Scientific, Waltham, MA, USA). Glass coverslips were closed with mowiol (Sigma-Aldrich, St. Louis, MO, USA).

Cryostat sections (10 μm) from muscle biopsies were incubated with the mouse-monoclonal primary antibody against SERCA1, followed by the Alexa Fluor 568 red secondary antibody.

Confocal microscopy was performed using a TCS-SP5 II confocal laser scanning microscope (Wetzlar, Germany).

### 4.5. Aequorin Ca^2+^ Measurements

HEK293 cells co-transfected with the cytosolic Ca^2+^ probe aequorin (cytAEQ) with empty and with WT, G211V, G211/286V, G286G SERCA1-expressing vectors in a 1:1 ratio, were pre-incubated for 1–3 h with 5 μM coelenterazine. Measurements and calibration of aequorin signal were performed as previously described [13].

### 4.6. Preparative Procedures and Biochemical Assay

All animal work was conducted according to the national and international guidelines for animal welfare. Semimembranosus muscle biopsy from G211V/G286V PMT-affected *Romagnola* cattle was collected from the Veterinary Clinic of the University of Bologna under local anesthesia during diagnostic procedures that would have been carried out anyway, as described in our previous work [3]. Control semimembranosus muscle samples were collected from healthy *Romagnola* animals of the same gender and age, euthanized at the slaughterhouse. Biopsies were homogenized in 10 mM HEPES, pH 7.4, 20 mM KCl supplemented with protease inhibitors (Sigma-Aldrich, St. Louis, MO, USA), as described in [8]. The myofibrils were sedimented by centrifugation at 650× *g* for 10 min at 4 °C. The crude SR fraction was obtained from the previous supernatant by ultracentrifugation at 120,000× *g* for 90 min at 4 °C. The final supernatant, representing the soluble sarcoplasm, was saved. Membrane fractions were resuspended in 0.3 M sucrose and 5 mM imidazole, pH 7.4, containing protease inhibitors and stored at −80 °C. The isolation of microsomal fraction from HEK293 cells was carried out according to Maruyama and MacLennan [11]. Briefly, at the end of incubation time, cells were washed with PBS and collected in 10 mM Tris-HCl pH 7.5 and 0.5 mM MgCl_2_ and homogenized with 30 strokes in a glass homogenizer. The homogenate was diluted with an equal volume of a solution of 10 mM Tris-HCl, pH 7.5, 0.5 M sucrose and 300 mM KCl. The suspension was centrifuged at 10,000× *g* for 30 min at 4 °C to pellet nuclei and mitochondria. The pellet was discarded, and the supernatant was further centrifuged at 100,000× *g* for 150 min at 4 °C to obtain the microsomal fraction. The final pellet was re-suspended in 0.25 M sucrose and 10 mM MOPS containing protease inhibitors. Protein concentrations were determined by the Bicinchoninic Protein Assay Kit.

ATPase activity of the SR microsomal fractions (20 μg/mL) was measured by spectrophotometric determination of NADH oxidation coupled to an ATP regenerating system, as previously described [8]. The assay was performed at 37 °C in the presence of 2 μg/mL A23187 Ca^2+^ ionophore at pCa5. For investigating the Ca^2+^ dependence of ATPase activity, the concentration of free Ca^2+^ was varied from pCa9 to pCa3 using EGTA-buffered solutions [8].

NADH coupled with the ATPase assay protocol adapted for use on a 96-well microplate reader was used to analyze the ATPase activity of the microsomal fraction from HEK293 cells (5 μg/well), as previously described [32]. The absorbance change at 340 nm was monitored using the EnVision (PerkinElmer, Waltham, MA, USA) plate reader. The experiments were performed at 37 °C in a final volume of 200 μL at pCa5 [21] on the same buffer described above. Technical duplicates were performed for each experiment.

### 4.7. Immunoprecipitation

HEK293 cells were washed with PBS and subsequently lysed in RIPA solubilization buffer (50 mM TRIS-HCl pH 7.4, 150 mM NaCl, 1% NP40, 0.5% sodium deoxycholate, 0.1% sodium dodecyl sulfate) supplemented with 2 mM *N*-ethyl-maleimide (NEM) (Sigma) and protease inhibitors. 450 μg of total-cell lysate (input) was subjected to immunoprecipitation. Samples were incubated overnight, tumbling at 4 °C with the polyclonal antibodies specific to SERCA1 (Cell Signaling, Danvers, MA, USA). The following day, protein G-magnetic beads (Millipore, Darmstadt, Germany) were added and incubated for 1 h at 4 °C. Beads were recovered, extensively washed with RIPA buffer containing *N*-ethyl-maleimide (NEM) and aspirated to dryness. SERCA1 was eluted by heating beads at 70 °C in sample-loading buffer with added beta-mercaptoethanol for subsequent detection by Western blotting. The blots were blocked with Tris-buffered saline, Tween 20 (TBS-T) with 5% milk powder and probed with mouse anti-ubiquitin (Merck Millipore, Burlington, MA, USA) and then incubated with secondary HRP-conjugated antibodies (Vector Laboratories, Newark, CA USA). Bands were detected as described above. Images were acquired by the Alliance mini HD9 Imaging System.

### 4.8. RNA Preparation and Quantitative Real-Time PCR

Total RNA extraction from semimembranosus muscle and cDNA synthesis were performed as previously described [4,8]. Real-time PCR was performed by the SYBR Green method with an Applied Biosystems 7500 Fast Real Time PCR System. Reaction conditions were as follows: 2 min at 50 °C, 10 s at 95 °C, and 40 cycles of 15 s at 95 °C, 1 min at 60 °C. Oligonucleotide primers used were: SERCA1 5′-GCACTCCAAGACCACAGAAGA-3′ (sense), 5′-GAGAAGGATCCGCACCAG-3′ (antisense); glyceraldehydes-3-phosphate dehydrogenase 5′-GGTCACCAGGGCTGCTTTTA-3′ (sense) and 5′-GAAGATGGTGATGGCCTTTCC-3′ (antisense).

### 4.9. Statistical Analysis

Data were expressed as means ± SD. Statistical differences among groups were determined by one-way ANOVA test, followed by Dunnett’s test for simultaneous multiple comparisons with control. When only two groups were considered, statistical analysis was performed by the unpaired two-tailed Student’s *t*-test. A level of confidence of *p* ≤ 0.05 was used for statistical significance.

## Data Availability

The bovine SERCA1 protein structure is originated from PDB ID 3TLM; SERCA sequences are from www.ncbi.nlm.nih.gov.

## Supplementary Materials

The supporting information can be downloaded at: https://www.mdpi.com/article/10.3390/ijms232012364/s1.
