# Supplementary material for: Differential Analysis of Gly211Val and Gly286Val Mutations Affecting Sarco(endo)plasmic Reticulum Ca2+-ATPase (SERCA1) in Congenital Pseudomyotonia Romagnola Cattle
Source: Int J Mol Sci. 2022 Oct 15;23(20):12364. doi: 10.3390/ijms232012364 (PMC9604440; doi:10.3390/ijms232012364)
Supplement: Supplementary file 1 [file ijms-23-12364-s001.zip › ijms-1883002-supplementary.pdf]

Supplementary Figures

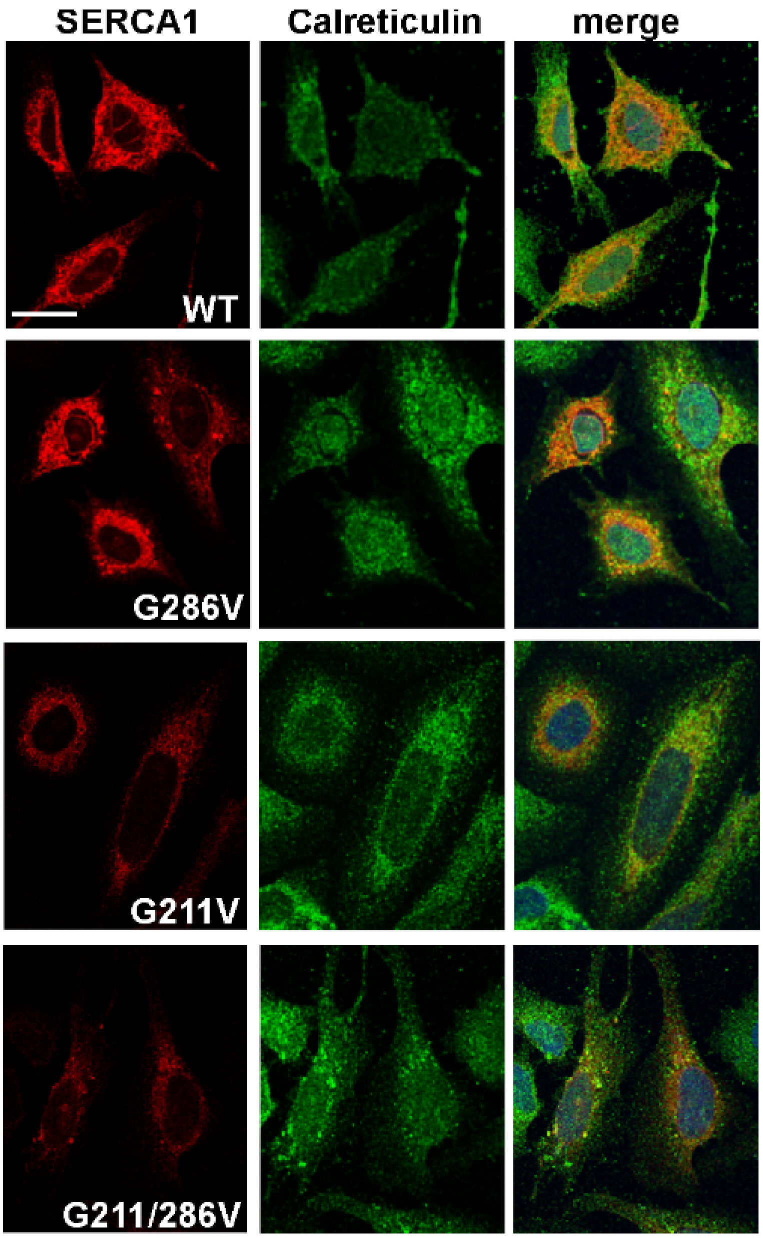

Figure S1. Localization of mutants SERCA1 to endoplasmic reticulum membranes. Cells immunolabelled with monoclonal antibodies to SERCA1 (red fluorescence) were subsequently incubated with polyclonal antibodies to calreticulin (green fluorescence) and then with the appropriate secondary antibody. The yellow signal resulting from the simultaneous visualization of the red and green fluorochromes, shows overlap of SERCA1 and ER marker. Nuclear morphology was demonstrated by staining with Hoechst. Images were recorded at the same setting conditions and magnification (scale bar 50 μm).

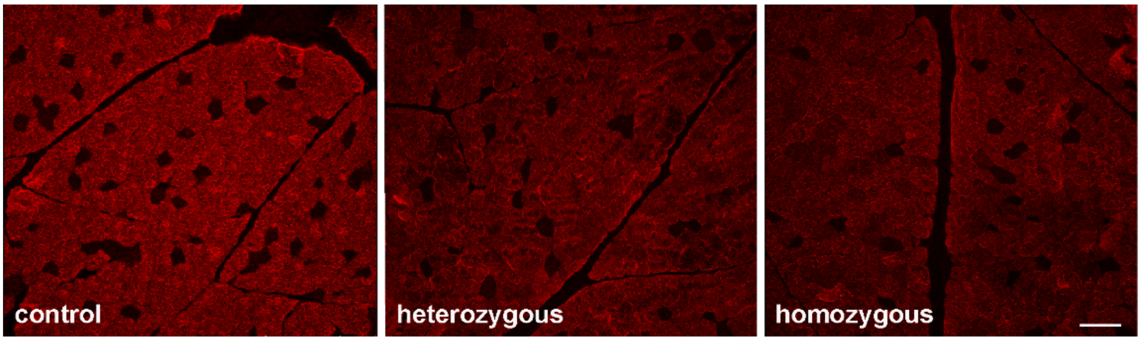

Figure S2. SERCA1 expression and localization in semimembranosus muscle cryosections from control and PMT affected Romagnola calves. Transverse sections from control healthy, heterozygous (G211V/G286V and R164H**)** and homozygous (G211V/G286V) PMT affected Romagnola calves were immunolabelled with monoclonal antibodies to SERCA1 isoform. Primary antibody was revealed by the secondary TRITC-conjugated antibody. Results showed that in all samples most of the fibers were labelled and only a small number of fibers were unstained with this antibody. Unstained fibers represent slow-twitch type 1 fibers expressing SERCA2 isoform, as already demonstrated in [8]. These results are in full agreement with the knowledge that bovine semimembranosus muscle is predominantly fast-twitch muscle, containing only 8-10% of slow-twitch fibers, based on the MHC isoforms composition. In both pathological muscle specimens, a reduction of immunoreactivity can be observed when compared with normal muscle. Images were recorded with a Leica laser scanning confocal microscope at the same setting conditions and magnification (scale bar 100 µm).

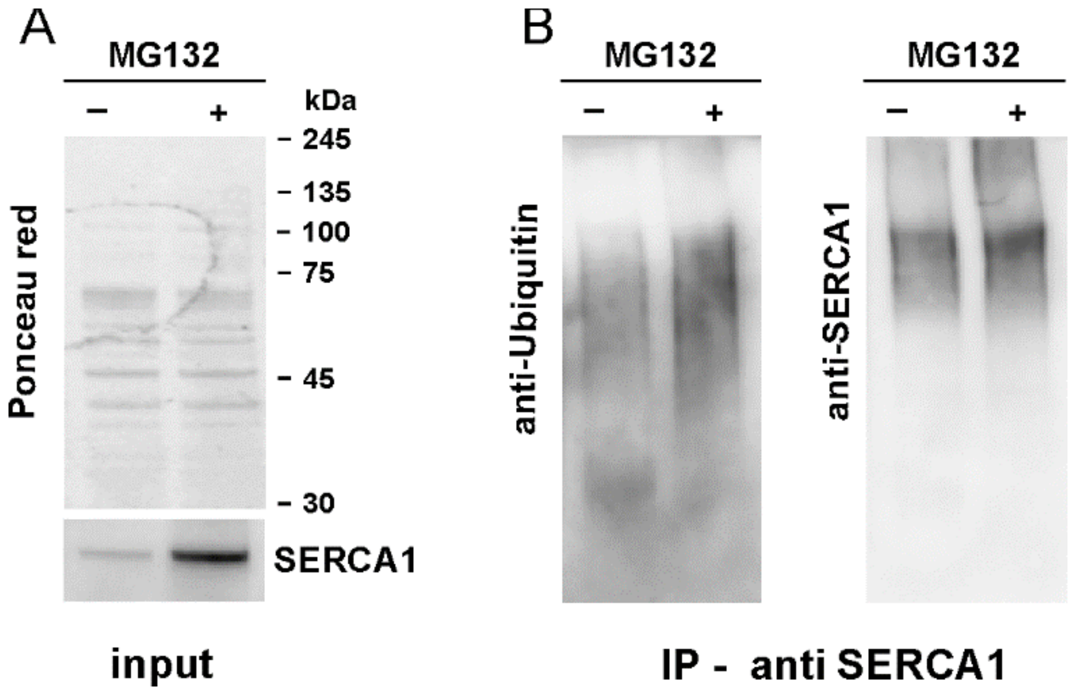

Figure S3. Polyubiquitination of G211V SERCA1 protein. HEK293 cells transfected with G211V SERCA1 cDNA were treated with the proteasome inhibitor MG132 (10 µM) (+) or its vehicle DMSO (0.1%) (-). Cells were lysed in RIPA buffer and 450 µg of total cell lysates (input) were used to immunoprecipitate SERCA1 with polyclonal antibodies. **A)** An equal quantity of protein from total cell lysates (input) was separated by SDS-PAGE and probed with antibodies specific to SERCA1. The upper panel shows Ponceau red staing. **B)** Immunocomplexes were resolved by SDS-PAGE. The ubiquitin-conjugated SERCA1 was tested with anti ubiquitin antibodies. Thereafter the antibodies were stripped from the filter and the paper was then probed with monoclonal antibodies to SERCA1.

Table S1. Mutations of the ATP2A1 gene, that have been detected in cattle breeds until now, are listed.

Cattle Breed	Genetic variant	Transcript change	Zygosity in cases	Exon	Protein sequence change	Protein sequence impact	Phenotype description
Chianina	g.25940510C>T	c.491G>A	Homozygous	Exon 6	R164H	Missense	Pseudomyotonia (PMT) [1, 5]
Romagnola	g.25939366C>A<br>g.25939141C>A	c.632G>T<br>c.857G>T	Homozygous	Exon 8	G211V<br>G286V	Missense	Pseudomyotonia (PMT) [3]
	As above	As above	Compound Heterozygous	Exon 6<br>Exon 8	G211/286V + R164H		
Dutch Improved Red and White	g.25933247G>A	c.1675C>T	Homozygous	Exon 14	R559C	Missense	Pseudomyotonia (PMT) [4]
Belgian Blue	g.25933247G>A	c.1675C>T	Homozygous	Exon 14	R559C	Missense	Congenital muscular dystonia 1 (CMD1) [2]
